# Decreased choroidal thickness in vitiligo patients

**DOI:** 10.1186/s12886-018-0796-0

**Published:** 2018-05-29

**Authors:** Serkan Demirkan, Zafer Onaran, Güzin Samav, Fatma Özkal, Erhan Yumuşak, Özgür Gündüz, Ayşe Karabulut

**Affiliations:** 1 Department of Dermatology and Venerology, Kirikkale University Faculty of Medicine, Yenisehir District, Tahsin Duru Avenue, No:14, Yahsihan, Kirikkale, Turkey; 2 Department of Ophtalmology, Kirikkale University Faculty of Medicine, Yenisehir District, Tahsin Duru Avenue, No:14, Yahsihan, Kirikkale, Turkey

**Keywords:** Vitiligo, Choroidal thickness, OCT, VASI, Oculocutaneous disease

## Abstract

**Background:**

Vitiligo is a disease characterized by depigmented macules and patches that occur as a result of the loss of functional melanocytes from the affected skin through a mechanism which has not been elucidated yet. Destruction of pigment cells in vitiligo may not remain limited to the skin; the eyelashes, iris, ciliary body, choroid, retinal pigment epithelium and meninges may also be affected. This study aims to compare the choroidal thickness of patients with and without vitiligo using optical coherence tomography (OCT).

**Methods:**

Spectral-domain optical coherence tomography (SD-OCT) (Retina Scan Advanced RS-3000 NIDEK, Japan) instrument (with λ = 840 nm, 27,000 A-scans/second and 5 μm axial resolution) was used for the imaging. Statistical analysis was performed using SPSS 21.0 software package.

**Results:**

In all values except optic nevre area measurements, the choroidal thickness of all vitiligo patients was found out to be thinner compared to the control group.

**Conclusions:**

In vitiligo, the choroidal thickness may be affected by the loss of melanocytes.

## Background

Vitiligo is a disease characterized by depigmented macules and patches that occur as a result of the loss of functional melanocytes from the affected skin through a mechanism which has not been elucidated yet. The frequency of vitiligo throughout the world changes in the rate of 0.5–2% and does not vary depending on gender and race [1–3]. While vitiligo may occur at all ages soon after birth, the average age of onset is approximately 20 years [1–3].

The choroid is a vascularized and pigmented tissue which was first examined histologically in the 17th century and then tried to be visualized by various methods [4]. The choroid of the eye is a highly vascularized structure that supplies the outer retina and, histologically, consists of a thin choriocapillaris layer that is adjacent to the retinal pigment epithelium (RPE) and Bruch’s membrane, medium- and large-caliber vessels (known as Sattler’s and Haller’s layers, respectively), and a suprachoroidal layer, all embedded within a collagenous and elastic stroma along with melanocytes [5]. The choroidal changes in many ocular pathological conditions such as polypoidal choroidal vasculopathy and age related macular degeneration were reported [6]. Choroidal thickness changes has also previously been observed in many systemic inflammatory disorders [6–9].

Melanocytes in the eyes consist of neural crest cells that have migrated ventrally. These melanocytes are located in the uveal tract (choroid, ciliary body, and the iris). Especially the stroma of the choroid layer consists of a high number of melanocytes [5]. The melanin, which is produced in melanocytes in the choroid layer, has an important function in an area starting from the retina and extending to the visual cortex of the brain. Melanin, which is produced in melanocytes in the eye and stored in melanosomes, has a very important role in the protection of the eye from the intraocular reflections of the light [5].

Destruction of pigment cells in vitiligo may not remain limited to the skin; the eyelashes, iris, ciliary body, choroid, retinal pigment epithelium and meninges may also be affected [10]. Low choroidal thickness may be expected in vitiligo where melanocyte loss proceeds [10].

Although there have been many studies conducted to evaluate choroidal thickening in diseases that affected eye vasculature, limited research has been conducted on the diseases that affect melanocytes and another component of choroidal tissue, which remained under-researched. This study aims to compare the choroidal thickness of patients with and without vitiligo using optical coherence tomography (OCT).

## Methods

This prospective clinical study addresses the examination of the bilateral eyes of (154 eyes). A total of 77 individuals, including 34 vitiligo and 43 non-vitiligo, were included in the study. This study was carried out between 2015 and 2016 in accordance with the tenets of the Declaration of Helsinki. The study protocol was approved by the Local Ethical Committee of the University of Kırıkkale. All patients and control subjects voluntarily participated in this study and signed an informed consent form.

Patients, who were diagnosed with vitiligo and were aged between 20 and 50 years, and non-vitiligo adults with similar characteristics participated in this study. VASI (vitiligo area severity index), which shows the depigmentation extent, was calculated in all vitiligo patients [11]. The percentage of the body area involved can be estimated by the so-called 1% rule or “palm method”. In both children and adults, the palm of the hand, including the fingers, is approximately 1% of the total body surface area (TBSA), and it describes hand unit [11]. For each body region, the VASI was determined by the product of the area of vitiligo in hand units and the extent of depigmentation within each hand unit–measured patch (possible values of 0, 10, 25, 50, 75, 90% or 100%). The total body VASI was calculated using the following formula considering the contributions of all body regions (possible range, 0–100):

$$ \mathrm{VASI}=\sum \mathrm{All}\ \mathrm{Body}\ \mathrm{Sites}\ \left[\mathrm{Hand}\ \mathrm{Units}\right]\times \left[\mathrm{Residual}\ \mathrm{Depigmentation}\right] $$

All participants had a thorough ophthalmologic examination, uncorrected visual acuity, best corrected visual acuity, manifest refraction, cycloplegic refraction and slit-lamp examination. Intraocular pressures were measured with an air-puff tonometer. Dilated fundus examinations were performed using a 78 D lens.

Individuals with poor OCT quality having a history that may have affected the choroidal thickness, such as diabetes, cigarette use, hypertension, antihypertensive drug use, known atherosclerotic disease, pregnancy, macular degenerations, previous ocular surgery, choroidal pathology, glaucoma, high refractive error (patients with more than + 6 and −6 diopters as cycloplegic spherical equivalent), best corrected visual acuity below 20/25, and patients with a systemic other disease were not included in this study. Spectral-domain optical coherence tomography (SD-OCT) (Retina Scan Advanced RS-3000 NIDEK, Japan) instrument (with λ = 840 nm, 27,000 A-scans/second and 5 μm axial resolution) was used for the imaging.

**Fig. 1:**
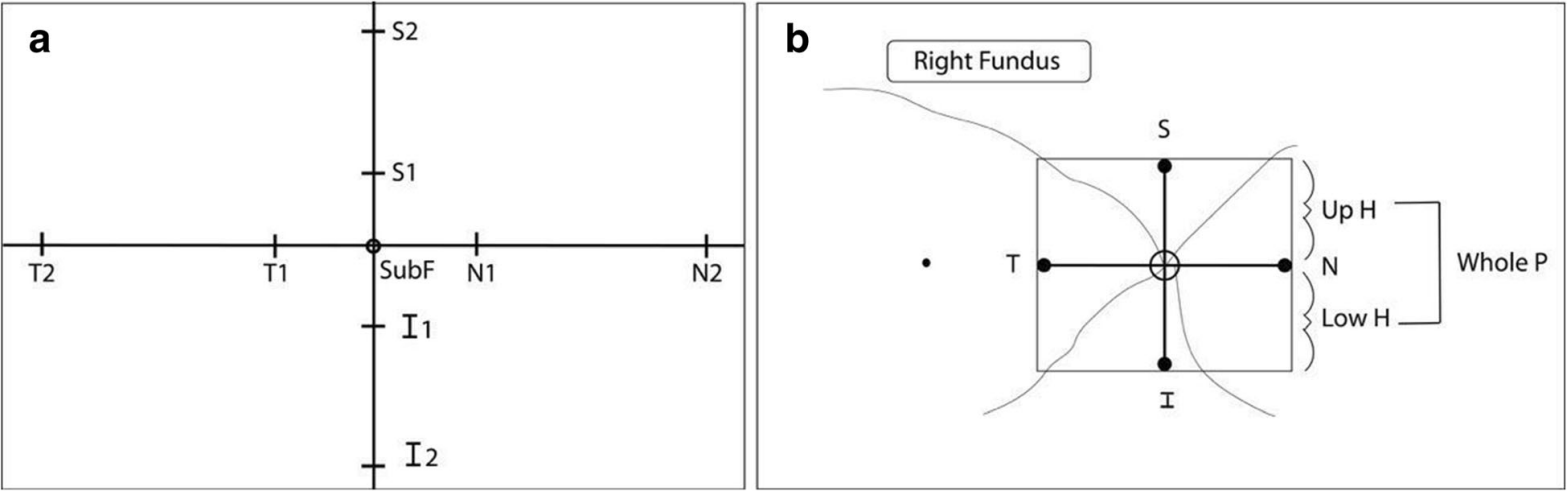
**a**, **b** The areas of choroidal thickness measurements

Before evaluation, using EDI-OCT scanning, the central macular thickness was measured in the right eye of each patient. Choroidal and scleral boundaries were drawn with the assistance of software programs. Choroidal thickness was measured at the center of the fovea (SubF), and 500 μm nasally, temporally, superiorly and inferiorly (N1, T1, S1, I1), and 1500 μm (N2, T2, S2, I2) from the center of the fovea. The peripapillary region was measured 500 μm (N, T, S, I) from the center of the optic nerve. The averages of upper hemifield, lower hemifield, and whole hemifield of the peripapillary region were also measured (Fig. 1a, b). The foveal and parafoveal choroidal thickness was determined by measuring the region between the outer border of the retinal pigment epithelium layer and the sclero-choroidal interface manually. Measurements in the peripapillary area were carried out automatically with the instrument. The values of the right and left eyes of the patient and control group were separately specified and compared. All measurements are presented with median, minimum and maximum values.

Statistical analysis was performed using SPSS 21.0 software package. Descriptive statistics were presented as a mean ± standard deviation. In comparisons between patient and control groups, the student’s t-test was applied to numerical data that followed a normal distribution, while the Mann-Whitney U test was applied to data that did not follow a normal distribution. The Pearson correlation test was applied to normally distributed measurements, and the Spearman correlation test was applied to data that did not follow a normal distribution. The statistical significance value was accepted as *p* < 0.05.

## Results

Thirty four vitiligo patients and 43 individuals without vitiligo diagnosis were included in the study. The mean age of the vitiligo patients was 39.2 years, and the average age of the individuals in the control group was 39.3 years. Table 1 shows the age and sex distrubation, intraocular pressure, axial length, visual acuity, and refraction defect values of the patients and control group.

**Table 1:** Age and sex distrubation, intraocular pressure, axial length, visual acuity, refraction defect values of the patients and control group

	Patients (n:34) (mean±)	Control group (n:43) (mean±)	*P* value
Age	39.2 ± 16.14	39.3 ± 12.51	0.101*
Sex(F/M)	15:19 (44%:56%)	20:23 (46%:54%)	
Right intraocular pressures	14.20 ± 3.31	15.00 ± 2.23	0.105*
Left intraocular pressures	14.55 ± 2.83	14.76 ± 2.09	0.347*
Axial length	23.57 ± 1.04	23.58 ± 1.22	0.960*
Right eyes visual acuity	0.07 ± 0.21	−0.01 ± 0.26	0.330*
Left eyes visual acuity	0.08 ± 0.22	0.06 ± 0.40	0.845*
Right eye refraction defect	− 0.37 ± 1.00	0.00 ± 0.92	0.184*
Left eyes refraction defect	−0.21 ± 0.97	− 0.01 ± 0.98	0.702*

*…Student’s t test

In all values except optic nerve area measurements, the choroidal thickness of all vitiligo patients was found out to be thinner compared to the control group (Table 2). Correlation between VASI values of vitiligo patients and age, duration of disease, and choroidal thickness were signed in Table 3.

**Table 2:** Mean choroidal thickness in vitiligo patients and control group individuals

	Patient (n:34)				Control (n:43)				*P* value
	Mean ± SD	Minimum	Median	Maximum	Mean ± SD	Minimum	Median	Maximum	
Right, SubF	220.2 ± 39.8	170	224	290	261.4 ± 31.1	168	256	305	< 0.001**
Right, N1	223.6 ± 42.1	163	220	276	258.4 ± 32.5	190	248	302	< 0.001**
Right, N2	226.0 ± 39.2	130	220	340	261.5 ± 37.4	200	265	361	< 0.001*
Right, T1	220.5 ± 39.9	143	224	303	257.9 ± 34.2	139	257.5	311	< 0.001**
Right, T2	225.2 ± 41.1	109	220	280	253.4 ± 32.3	200	250	327	0.001*
Right, S1	222.9 ± 44.6	142	219	296	268.7 ± 38.1	198	271	289	< 0.001**
Right, S2	217.8 ± 40.8	151	219	301	259.1 ± 33.4	201	261	306	< 0.001**
Right, I1	223.4 ± 45.2	119	221	289	266.3 ± 37.0	136	264	321	< 0.001**
Right, I2	224.5 ± 45.2	117	226	340	265.9 ± 34.9	200	260	360	< 0.001*
Right optic nerve, LowH	95.0 ± 15.4	34	96	138	97.5 ± 7.9	80	97	118	0.429*
Right optic nerve, UpH	80.2 ± 16.8	43	81	128	77.1 ± 16.8	41	79	135	0.418**
Right optic nerve, WholP	98.9 ± 15.2	47	97	134	100.5 ± 8.1	54	101	126	0.805*
Right optic nerve, N	80.2 ± 16.8	54	77.5	126	77.1 ± 16.8	33	75	117	0.418**
Right optic nerve, T	68.9 ± 14.1	45	68	104	68.4 ± 15.5	29	69	99	0.689*
Right optic nerve, S	128.9 ± 25.3	59	132	168	129.4 ± 16.5	95	129	175	0.712*
Right optic nerve, I	118.9 ± 23.6	21	125.5	175	125.6 ± 17.0	79	125	165	0.230*
Left, SubF	222.7 ± 37.3	118	223	296	269.1 ± 31.0	129	267	305	< 0.001**
Left, N1	223.7 ± 38.5	105	224	301	271.7 ± 36.1	119	269	301	< 0.001**
Left, N2	228.2 ± 39.6	106	227	298	265.4 ± 35.8	129	267	311	< 0.001**
Left, T1	227.2 ± 42.2	164	226.5	380	308.2 ± 30.8	210	265	291	< 0.001*
Left, T2	235.4 ± 38.3	131	234	324	272.7 ± 35.7	176	275	329	< 0.001**
Left, S1	215.3 ± 40.2	126	216	305	257.2 ± 35.2	161	254	298	< 0.001**
Left, S2	220.0 ± 37.6	137	219	299	249.5 ± 48.2	148	251	324	0.005**
Left, I1	216.5 ± 38.0	139	218	301	262.3 ± 31.7	167	264	341	< 0.001**
Left, I2	222.2 ± 37.1	170	220	344	262.8 ± 31.9	210	260	350	< 0.001*
Left optic nerve, LowH	94.8 ± 12.2	59	95	141	95.5 ± 11.0	48	97	173	0.955*
Left optic nerve, UpH	122.6 ± 19.5	75	96	124	127.3 ± 16.3	77	93	125	0.255**
Left optic nerve, WholP	98.1 ± 12.0	70	99	125	100.2 ± 11.7	79	101	133	0.655*
Left optic nerve, N	75.6 ± 21.6	30	78	126	82.1 ± 23.7	27	78	174	0.432*
Left optic nerve, T	66.0 ± 18.1	35	61.5	106	65.0 ± 14.3	38	66	95	0.782*
Left optic nerve, S	128.4 ± 19.9	81	131.5	162	128.6 ± 20.5	76	131	178	0.951*
Left optic nerve, I	122.6 ± 19.5	89	122	169	127.3 ± 16.3	97	127	174	0.310*

*…Mann Whitney U test

**…Student’s t test

**Table 3:** Correlation between VASI values of vitiligo patients and age, duration of disease, and choroidal thickness

	VASI	r	*p*
Age	Weak correlation	0.349^a^	0.043
Duration of disease	Moderate correlation	0.555^a^	< 0.001
Right fovea, horizontal	Negative correlation	−0.417^a^	0.014
Right nasal 500	Negative correlation	− 0561^a^	0.001
Right nasal 1500	Negative correlation	−0.381^b^	0.026
Left fovea, vertical	Negative correlation	−0.437^a^	0.010
Left superior 500	Negative correlation	−0.481^a^	0.004
Left inferior 500	Negative correlation	−0.484^a^	0.004
Left superior 1500	Negative correlation	−0.356^a^	0.039
Left inferior 1500	Negative correlation	−0.380^a^	0.027

^a^…Pearson correlation test

^b^Spearman correlation test

There was a negative correlation between age and choroidal thickness in some areas in patients. In patients and control groups, gender had an effect on the choroidal difference in none of the measured regions (*p* > 0.05). There was no correlation between duration of disease and choroidal thickness in all areas.

In those with higher VASI value, periorbital involvement was significantly more frequent. (*p* = 0.029). The frequency of periorbital involvement increased with the duration of the disease (*p* < 0.001). The periorbital involvement did not have an effect on choroidal thickness in patients with vitiligo. There was no statistically significant difference between those with and without periorbital involvement concerning age (*p* = 0.300).

## Discussion

The stroma of the choroid layer consists of a high number of melanocytes [5]. Destruction of pigment cells in vitiligo may not remain limited to the skin; the eyelashes, iris, ciliary body, choroid, retinal pigment epithelium and meninges may also be affected [10]. A low choroidal thickness may be expected in vitiligo where melanocyte loss proceeds [10]. To our knowledge, this is the first study that examined the relation between choroidal changes and vitiligo in adulthood.

The choroid covers the outer retina and is among the most vascularized tissues in the body. This tissue supplies oxygen and nutrition to and provides temperature regulation for the retina. Also, choroid-containing melanocytes prevent intraocular reflections. In the eye, choroidal thickness may be affected by several factors, such as age, axial length, and refractive errors [12, 13]. A number of studies have found that choroidal thickness plays a prognostic or predictive role in various local (e.g., diabetic retinopathy), and systemic diseases (e.g., hypertension, anemia, rheumatoid arthritis and obesity) [14–20].

In oculocutaneous albinism patients with melanocyte absence, the choroidal thickness in the subfoveal area was found to be significantly lower compared to the control group. However, no difference was found in the peripapillary region compared to the control group [21]. Choroidal thickness measurement was compared in a much higher number of regions in our study compared to the aforementioned study in which the lower choroidal thickness is also expected in vitiligo, which is another disease that proceeds with melanocyte loss [21].

Vogt-Koyanagi-Harada Diseaseis a bilateral granulomatous panuveitis associated with autoimmunity developed against melanocytes [22]. Patients with VKH increased choroidal thickness, which is probably due to exudation with inflammatory processes [23]. Invitiligo patients, the inflammatory process is chronic and exudative is not observed. Therefore, despite the presence of melanocyte destruction as it is in VKH, the increase in choroidal thickness of vitiligo patients is not expected.

The study conducted by Bulbul-Baskan et al. showed that eye pathology was observed in 10 of the 45 vitiligo patients. Their findings revealed that iris involvement in one patient, ring-like peripapillary atrophy around the optic nerve in seven patients, hyperpigmented rim in the left top segment of the retinal pigment epithelium in addition to peripapillary atrophy in one patient, focal hypopigmented dots in the temporal retinal area in one patient, and diffuse hypopigmentation in onepatient were observed [24]. Another study carried out with black patients with vitiligo, thin and dot-like pigmentary disturbances were identified in four of the 17 patients [25].

In the current study, we observed a significant reduction in OCT in all areas except optic nerve regions in the vitiligo patients. When we reviewed the relevant literature on this subject, we have not seen any published studies that would allow us to make a direct comparison regarding our findings. The lack of differences between the vitiligo patients and the control group in optic nerve regions may be because melanocytes occupy less space in the histological structure in the optic nerve regions.

Some studies maintained that gender and hormonal status may influence choroidal blood flow and lead to change in the choroidal thickness [26, 27]. However, in our study, it was observed that gender resulted the difference in choroidal thickness neither in the vitiligo patients group nor the control group.

Many authors have reported that the reasons for the differences in the choroidal thickness results between studies are different software programs for measurement, differences in the light source of the OCT, ethnic differences, differences in the age, refraction defects and axial length in the patient profile [14–20]. However, since a comparison was made with the control group, and the characteristics of the patient and control group were similar, the findings suggest that comparison of the measurements resulted in useful data.

## Conclusion

Melanin, which is produced in melanocytes in the eye and stored in melanosomes, has a very important role in the protection of the eye from the intraocular reflections of light. In this study, in all values except optic nerve area measurements, the choroidal thickness of all vitiligo patients was found out to be thinner compared to the control group.

The melanocyte amount in the choroidal layer in vitiligo should be studied in the future postmortem and in vivo studies.

## Abbreviations

CT: Choroidal thickness
EDI-OCT: Enhanced-depth imaging optical coherence tomography
I: Choroidal thickness at 500 μm inferior to the fovea
I1: Choroidal thickness at 500 μm inferior to the fovea
I2: Choroidal thickness at 1500 μm inferior to the fovea
LowH: LowerHemifield
N: Choroidal thickness at 500 μm nasal to the fovea
N1: Choroidal thickness at 500 μm nasal to the fovea
N2: Choroidal thickness at 1500 μm nasal to the fovea
OCT: Optical coherence tomography
RPE: Retinal pigment epithelium
S: Choroidal thickness at 500 μm superior to the fovea
S1: Choroidal thickness at 500 μm superior to the fovea
S2: Choroidal thickness at 1500 μm superior to the fovea
SD-OCT: Spectral-domain optical coherence tomography
SubF: Choroidal thickness at fovea
T: Choroidal thickness at 500 μm temporal to the fovea
T1: Choroidal thickness at 500 μm temporal to the fovea
T2: Choroidal thickness at 1500 μm temporal to the fovea
UpH: Upper Hemifield
VASI: Vitiligo area severity index
VKH: Vogt-Koyanagi-Harada
WholP: Whole peripapillary
